# Precision in Pediatric Obstructive Sleep Apnea: Preventing Unnecessary Surgery Using Drug-Induced Sleep Endoscopy

**DOI:** 10.7759/cureus.111034

**Published:** 2026-06-17

**Authors:** Sofia Freitas, Ana S Cruz, Isabel E Costa, Petra Alves, Cristina Gomes

**Affiliations:** 1 Anesthesiology, Unidade Local de Saúde de Braga, Braga, PRT; 2 Otolaryngology - Head and Neck Surgery, Unidade Local de Saúde de Braga, Braga, PRT

**Keywords:** adenotonsillectomy, airway obstruction, drug-induced sleep endoscopy, epiglottic collapse, obstructive sleep apnea, pediatric airway, sleep endoscopy

## Abstract

Pediatric obstructive sleep apnea (OSA) is often multifactorial, and awake airway assessment may fail to accurately identify the sites of upper airway obstruction. Drug-induced sleep endoscopy (DISE) enables dynamic evaluation of the upper airway under sleep-like conditions and may be particularly valuable in children at an increased risk of persistent OSA after adenotonsillectomy.

We report the case of a 10-year-old girl with severe OSA, obesity (body mass index: 31.04 kg/m², above the 99th percentile), and neurocognitive impairment, in whom awake assessment suggested adenotonsillar hypertrophy as the presumed primary contributor to obstruction. During sleep-state evaluation, DISE demonstrated isolated positional epiglottic collapse as the dominant dynamic finding, refining the assessment of the obstruction pattern and supporting avoidance of adenotonsillectomy.

This case highlights the role of DISE in guiding individualized, anatomy-based management of pediatric OSA and emphasizes the importance of carefully titrated anesthesia to ensure accurate assessment of airway dynamics.

## Introduction

Pediatric obstructive sleep apnea (OSA) affects 1% to 4% of children and is associated with significant neurobehavioral, cardiovascular, and metabolic morbidity if left untreated [1]. Overnight polysomnography remains the diagnostic gold standard; however, it does not provide information on the anatomical sites of airway obstruction [1]. Adenotonsillar hypertrophy is the most common cause in otherwise healthy children, yet residual or persistent OSA after adenotonsillectomy is reported in up to one-third of cases, with higher rates among children with obesity, craniofacial anomalies, neuromuscular disorders, or neurodevelopmental impairment [2,3]. These risk factors may reduce the predictive value of awake anatomical examination alone and support a more individualized preoperative assessment in selected patients.

These limitations highlight the need for precise identification of obstruction sites to optimize treatment selection. Drug-induced sleep endoscopy (DISE) has therefore emerged as a valuable diagnostic modality, enabling real-time dynamic evaluation of airway collapse under pharmacologically induced sleep and supporting tailored, anatomy-based management, particularly in high-risk or clinically discordant cases [4-6]. DISE interpretation may vary across institutions due to differences in scoring systems, anatomical sites assessed, definitions of collapse patterns, and anesthetic protocols [5,7,8]. These limitations should be considered when interpreting DISE findings, but they do not preclude its clinical value in selected patients in whom dynamic airway assessment may change management.

Within this context, we report the case of a child with severe OSA, obesity, and neurocognitive impairment in whom DISE refined the preoperative assessment by identifying isolated positional epiglottic collapse as the dominant dynamic finding, resulting in a change in the management and supporting avoidance of adenotonsillectomy.

## Case presentation

A 10-year-old girl weighing 77.5 kg, with a body mass index of 31.04 kg/m², above the 99th percentile for age and sex, neurocognitive impairment, and chronic oral corticosteroid therapy for atopic dermatitis, presented with habitual snoring, witnessed apneas, and excessive daytime somnolence. Baseline polysomnography confirmed severe OSA, with an obstructive apnea-hypopnea index of 15 events/hour and an oxygen saturation nadir of 85%.

Awake airway evaluation suggested inferior turbinate hypertrophy, grade II adenotonsillar hypertrophy, and a Mallampati class III airway. Adenotonsillectomy with inferior turbinate reduction was initially proposed. However, given the presence of obesity and developmental delay, which are recognized risk factors for persistent postoperative OSA, further evaluation with DISE was performed before surgery.

Anesthetic technique

Sedation aimed to reproduce sleep-like upper airway conditions while preserving spontaneous ventilation. After premedication with oral midazolam 10 mg, brief inhalational induction with sevoflurane was used solely to facilitate intravenous access, after which the volatile agent was discontinued. Propofol was then administered by target-controlled infusion using the Paedfusor model, starting at 1.0 μg/mL and titrated according to clinical effect.

Sedation depth was guided by clinical assessment, with bispectral index (BIS) monitoring used as an adjunctive parameter rather than as a predefined sedation target. Cerebral oximetry was used as adjunctive monitoring during obstructive events and to support the interpretation of associated cerebral oxygenation changes. Spontaneous ventilation was maintained throughout the procedure, and topical anesthetics, nasal decongestants, and airway maneuvers were avoided to minimize alteration of upper airway dynamics. 

At an observed BIS value of 67, adequate sleep-like conditions with reproducible snoring and preserved spontaneous ventilation were achieved, allowing DISE assessment. A brief transient oxygen desaturation to 74% occurred and resolved spontaneously without assisted ventilation or airway rescue maneuvers.

DISE findings

Airway evaluation was performed systematically using the velum, oropharyngeal lateral walls, tongue base, and epiglottis (VOTE) classification. No collapse was observed at the velopharyngeal or oropharyngeal levels, and the lateral pharyngeal walls remained patent. The tongue base was enlarged but non-obstructive.

**Figure 1:**
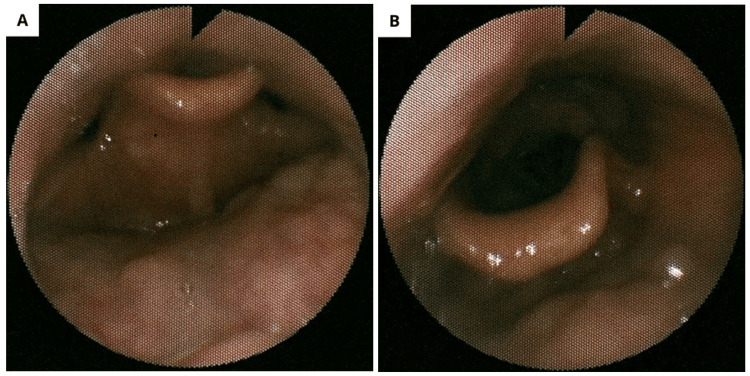
Dynamic epiglottic collapse during drug-induced sleep endoscopy (A) Isolated posterior trapdoor collapse of the epiglottis causing significant supraglottic obstruction. (B) Resolution of the obstruction following lateral head rotation. Written informed consent for publication of the clinical information was obtained from the patient’s legal guardian using the institutional consent form before submission. No identifiable patient information is included in the manuscript.

In contrast, a marked, isolated posterior trapdoor collapse of the epiglottis was observed, consistent with dynamic supraglottic obstruction. Diagnostic positional maneuvers, including gentle manual mandibular advancement under continuous endoscopic visualization, were then performed during endoscopic assessment to characterize the obstruction pattern. This collapse did not improve with mandibular advancement but resolved completely with lateral head rotation, supporting a positional component. These findings were discordant with the awake airway assessment and suggested that adenotonsillar hypertrophy was not the primary contributor to airway obstruction. Representative still images obtained during DISE demonstrated dynamic epiglottic collapse (Figure 1), while the corresponding findings according to the VOTE classification are summarized in Table 1.

**Table 1:** Drug-induced sleep endoscopy findings according to the VOTE classification VOTE: velum, oropharyngeal lateral walls, tongue base, and epiglottis

VOTE structure	Finding
Velum	No collapse observed
Oropharyngeal lateral walls	No collapse observed
Tongue base	No collapse observed
Epiglottis	Primary trapdoor collapse

Management 

Based on DISE findings showing no clinically significant adenotonsillar obstruction, adenotonsillectomy was not performed, and the initial surgical plan was revised. Given the presence of nasal obstruction, bilateral inferior turbinate reduction was performed during the same anesthetic and surgical session, immediately after completion of DISE.

The isolated positional epiglottic collapse identified during DISE was managed conservatively, with emphasis on positional therapy and referral for weight-management intervention. 

Outcome and follow-up 

The patient was scheduled for clinical follow-up to reassess the symptoms and treatment response. Conservative measures included daily nasal irrigation, sleep hygiene recommendations, such as avoiding screen exposure before bedtime, and an individualized nutritional plan.

At one-year follow-up, repeat polysomnography was not available; therefore, outcome assessment was based on clinical follow-up. The patient showed partial improvement in snoring and daytime somnolence, without reported complications. Residual symptoms were considered potentially multifactorial. Ongoing management focused on weight reduction and conservative measures, with consideration of further interventions, including positive airway pressure therapy, if clinically indicated.

## Discussion

This case highlights the limitations of awake airway assessment in pediatric OSA and demonstrates how DISE can directly influence clinical decision-making. In children with obesity and neurocognitive impairment, airway obstruction can be complex and dynamic and may not be accurately predicted by awake examination alone [2,4]. Previous studies have shown poor correlation between awake findings and sleep-state obstruction, with DISE identifying clinically relevant sites of collapse that may otherwise remain undetected [4,7]. These findings support the use of DISE in selected high-risk patients, particularly when the severity of OSA documented on polysomnography is not fully explained by awake upper airway examination.

Epiglottic/supraglottic collapse is a recognized finding assessed during pediatric sleep endoscopy and may be clinically relevant in selected patients with OSA [7,8]. In this case, isolated positional epiglottic collapse was identified as the primary mechanism of obstruction, a finding that would not have been addressed by adenotonsillectomy. Recognition of this pattern is important, as it may be managed with non-surgical strategies such as positional therapy, positive airway pressure therapy, or in selected cases, targeted surgical intervention. 

Importantly, DISE findings directly influenced surgical planning. Although awake examination showed grade II adenotonsillar hypertrophy, DISE did not demonstrate clinically significant dynamic obstruction at the adenotonsillar level; instead, isolated positional epiglottic collapse was identified as the predominant obstruction pattern. Therefore, adenotonsillectomy was deferred, and management was redirected toward nasal obstruction and conservative treatment of the positional epiglottic collapse. This is consistent with pediatric DISE literature showing that DISE can modify surgical planning and influence management strategies in children with OSA [2,6,9].

The accuracy of DISE findings is critically dependent on anesthetic management, as both the choice of sedative agent and the depth of sedation can significantly influence upper airway behavior and potentially lead to misinterpretation of obstruction sites [2,5]. Minimizing topical anesthetics may be beneficial because they can alter upper airway muscle activity and affect the assessment of airway dynamics [10]. In this patient, the anesthetic protocol was chosen to balance procedural tolerability and diagnostic validity: brief inhalational induction facilitated intravenous access in the setting of limited cooperation, while subsequent propofol target-controlled infusion allowed titratable sedation under institutional practice, with preserved spontaneous ventilation and minimal distortion of upper airway dynamics.

Despite its advantages, DISE has limitations, including variability in scoring systems, interobserver interpretation, and the fact that drug-induced sedation does not fully replicate natural sleep. Further standardization of technique, sedation protocols, scoring systems, and reporting is needed to optimize its role in clinical practice.

## Conclusions

This case highlights the limitations of awake airway assessment in pediatric OSA and demonstrates the value of DISE in identifying clinically relevant sites of obstruction. In this patient, DISE identified isolated positional epiglottic collapse as the predominant obstruction pattern, supporting avoidance of adenotonsillectomy and redirecting management toward individualized, anatomy-based treatment. The coexistence of obesity and neurocognitive impairment added complexity to airway assessment and supported the value of a DISE-guided approach. Careful anesthetic titration remains essential to preserve spontaneous ventilation and minimize distortion of upper airway dynamics during DISE.
